# Enhancing renal stone detection through artificial intelligence models

**DOI:** 10.3389/fmed.2026.1977903

**Published:** 2026-10-27

**Authors:** Xue Yang, Jian Kang, Jirui Niu, Li Ma, Yin Zhang, Zipu Dong

**Affiliations:** 1 Blood Sample Collection, The Fourth Affiliated Hospital of Harbin Medical University, Harbin, Heilongjiang, China; 2 Department of Urology, Nangang Branch, Heilongjiang Provincial Hospital and Harbin Institute of Technology, Harbin, Heilongjiang, China; 3 Department of General Surgery, The Fourth Affiliated Hospital of Harbin Medical University, Harbin, Heilongjiang, China; 4 Bio-Bank of Department of General Surgery, The Fourth Affiliated Hospital of Harbin Medical University, Harbin, Heilongjiang, China; 5 Ward 2, Department of Urology, Heilongjiang Provincial Hospital and Harbin Institute of Technology, Harbin, Heilongjiang, China; 6 Heilongjiang Nursing College, Harbin, Heilongjiang, China; 7 Department of Ultrasound, Heilongjiang Provincial Hospital & Harbin Institute of Technology, Harbin, Heilongjiang, China; 8 Department of Gastroenterological Surgery, Harbin Medical University Cancer Hospital, Harbin Medical University, Harbin, Heilongjiang, China; 9 Ward 1, Department of Urology, Heilongjiang Provincial Hospital and Harbin Institute of Technology, Harbin, Heilongjiang, China

**Keywords:** AI, artificial intelligence, HRNet, renal stone, ultrasound

## Abstract

**Objective:**

Traditional ultrasonic detection for kidney stones is prone to variability due to the experience and skill of the operating physician, leading to inconsistent diagnostic outcomes. Factors such as stone size, location, and surrounding tissues can further compromise accuracy. Artificial intelligence (AI), with its advanced data processing and deep learning capabilities, offers a solution by automating the analysis of medical images to improve diagnostic accuracy and efficiency.

**Methods:**

This cross-sectional study was conducted at our hospital between 01/06/2022 and 03/07/2024. After excluding 50 poor-quality ultrasound images, the final dataset included 1,130 images: 918 stone images and 212 non-stone images. At the patient level, 791 stone images were used for training/validation, and 127 stone images plus 212 non-stone images (*n* = 339) were held out as an independent test cohort. The High-Resolution Network (HRNet) was employed for model training to detect kidney stones in ultrasound images. Detailed image annotations were created using the labelme tool and converted to COCO format for compatibility with AI algorithms.

**Results:**

In the independent test cohort (*n* = 339), the confusion matrix was: TN = 200, FP = 12, FN = 34, and TP = 93, corresponding to 212 actual negatives and 127 actual positives. The HRNet model achieved an accuracy of 86.4% (95% CI 82.4–89.7%), sensitivity of 73.2% (95% CI 64.9–82.2%), specificity of 94.3% (95% CI 90.4–96.7%), positive predictive value (PPV) of 88.6% (95% CI 81.1–93.3%), and negative predictive value (NPV) of 85.5% (95% CI 80.4–89.4%). The model showed high specificity but moderate sensitivity, missing approximately 26.8% of renal stones in the test cohort.

**Conclusions:**

HRNet-based AI may improve specificity and reduce false-positive findings in ultrasound-based renal stone detection. However, the moderate sensitivity indicates a substantial risk of missed stones, and the model requires further optimization before clinical adoption. This retrospective study evaluated image-level performance only and did not directly measure patient outcomes, clinical workload, diagnostic time, or treatment decisions. Therefore, claims that this approach reduces diagnostic burden or improves patient outcomes are not supported by the present data.

## Introduction

Renal stone (RS) is one of the most common urological diseases (1, 2). The incidence of RS has increased dramatically in the last few decades (3). Ultrasound detection is one of the most commonly employed methods for diagnosing RS (4, 5). Its non-invasive nature and real-time imaging capabilities make it a preferred choice in clinical settings (6, 7). However, this technique is not without its limitations. The accuracy of ultrasound results is often heavily influenced by the experience and skill level of the operating physician (1–3, 8). Variations in interpreting ultrasound images among different doctors can lead to inconsistent diagnostic outcomes, which may significantly impact patient care (9). Moreover, the precision of ultrasound detection can be compromised by factors such as the size, location, and surrounding tissues of the stone (10, 11). These factors can obscure the stone or make it difficult to differentiate from other structures, thereby affecting the reliability of the diagnosis (12). Additionally, ultrasound detection demands prolonged concentration from doctors, which can easily result in fatigue and misjudgment, further complicating the diagnostic process (4, 6, 13).

**Table 1:** Dataset composition.

Group	Stone images	Non-stone images	Total images	Use
Train/val	791	0	791	Model development
Independent test	127	212	339	Final evaluation
Total	1,257	212	1,469	

With the rapid advancement of technology, artificial intelligence (AI) has emerged as a novel and powerful tool in medical diagnostics (14–16). AI, particularly through its robust data processing capabilities and deep learning algorithms, offers a promising solution to many of the challenges faced in traditional ultrasound detection of renal stones (17, 18). By training and refining algorithms, AI models can autonomously analyze medical images, pinpoint lesions, and enhance both the accuracy and efficiency of diagnoses (8, 16, 17, 19). This not only accelerates the diagnostic process for doctors but also alleviates their workload, ultimately delivering superior medical services to patients. The significance of AI in medical research is becoming increasingly pronounced, especially in the realm of kidney stone detection.

In recent years, a growing number of researchers have begun exploring the application of AI technology for the detection of renal stones. Initial studies have shown promising results, demonstrating that AI models can successfully implement automated analysis of ultrasound images and identify renal stones with high accuracy. These models are adept at extracting relevant feature information from images and classifying and identifying features through sophisticated algorithms, thereby facilitating the automatic detection of renal stones. The ability of AI to process large datasets and identify patterns that may be imperceptible to the human eye makes it a valuable tool in improving diagnostic outcomes.

However, the integration of AI into the domain of automatic renal stone detection is not without its challenges (20, 21). One of the primary issues is the quality and quantity of data required for training and refining these models. Large-scale, annotated datasets are essential to facilitate effective learning and validation of AI algorithms. The development of such datasets requires significant resources and collaboration between medical professionals and data scientists. Additionally, the reliability and stability of AI models must undergo rigorous testing and assessment to ensure their efficacy in real-world applications. Ensuring that AI models can consistently and accurately detect renal stones across a wide range of patient demographics and clinical scenarios is crucial for their widespread adoption.

To address these challenges and capitalize on the potential of AI, we have incorporated AI technology into the application research of ultrasonic detection for renal stones. Our study aims to develop and validate an AI model that can autonomously analyze ultrasound images to detect renal stones. By leveraging advanced data processing techniques and state-of-the-art AI algorithms, we hope to overcome the limitations of traditional ultrasound detection and provide a more reliable and efficient diagnostic tool for clinicians. This research focused on image-level detection performance. Clinical workload, diagnostic time, treatment decisions, and patient outcomes were not evaluated.

## Methods

### Study design and population

A cross-sectional study was conducted at Heilongjiang Provincial Hospital between 01/06/2022 and 03/07/2024. The training/validation set consisted of 791 stone images. The independent test cohort consisted of 127 stone images and 212 non-stone images (*n* = 339). The study population included adult patients who had been diagnosed with at least one renal stone via ultrasound imaging. Ultrasound examinations were performed as part of routine clinical care. The study aimed to evaluate the effectiveness of an AI model in detecting renal stones using ultrasound images. Figures 1, 2 illustrate the AI model process and the detailed methodology flowchart, respectively. The requirement for obtaining signed informed consent from participants or their guardians was waived due to the retrospective nature of the study and the use of anonymous data.

**Figure 1:**
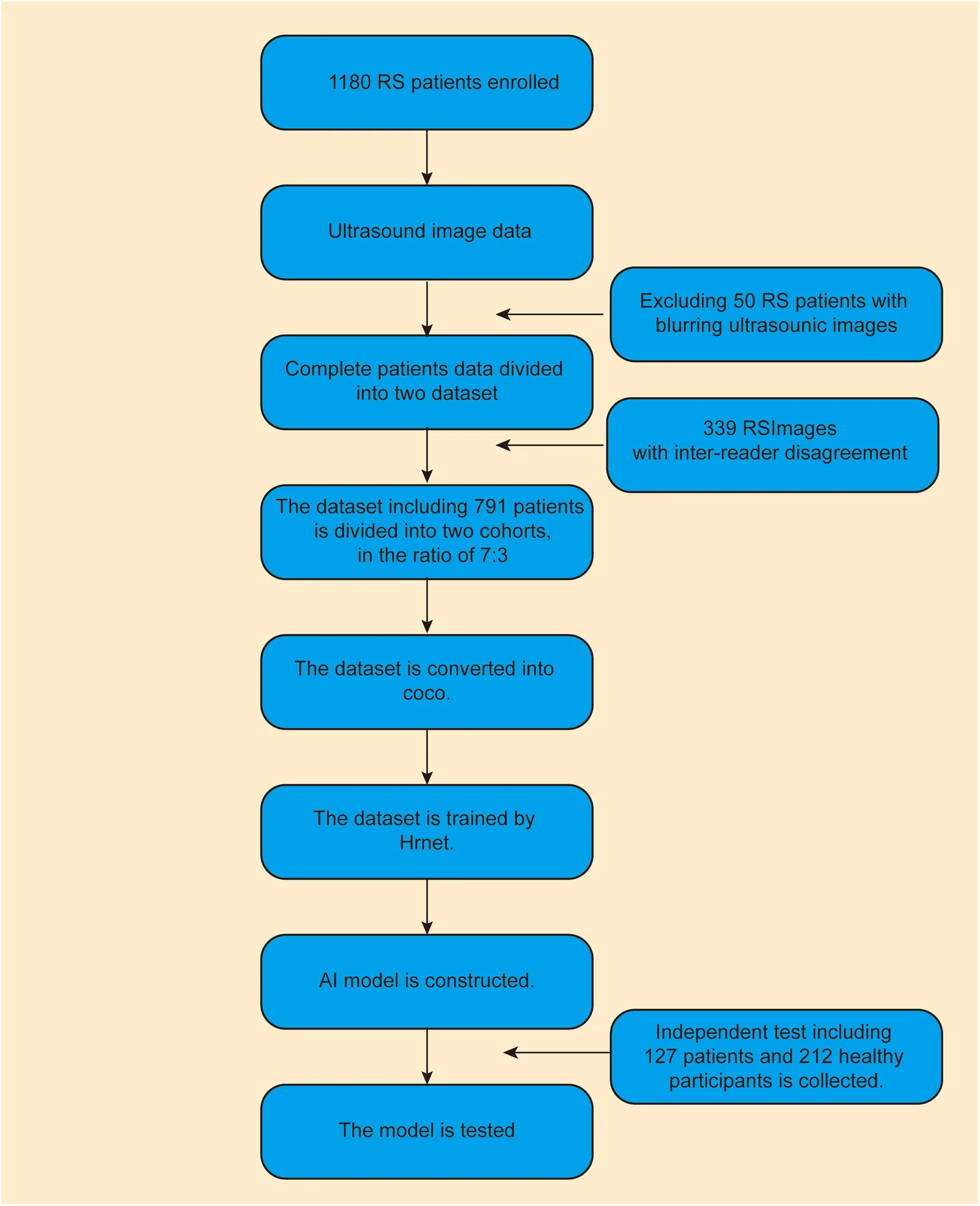
The overall flow chart of the model. Overall flow chart of the model. Initially, 1,180 participants were enrolled. After excluding 50 cases with poor ultrasound quality, 1,130 images were audited.

**Figure 2:**
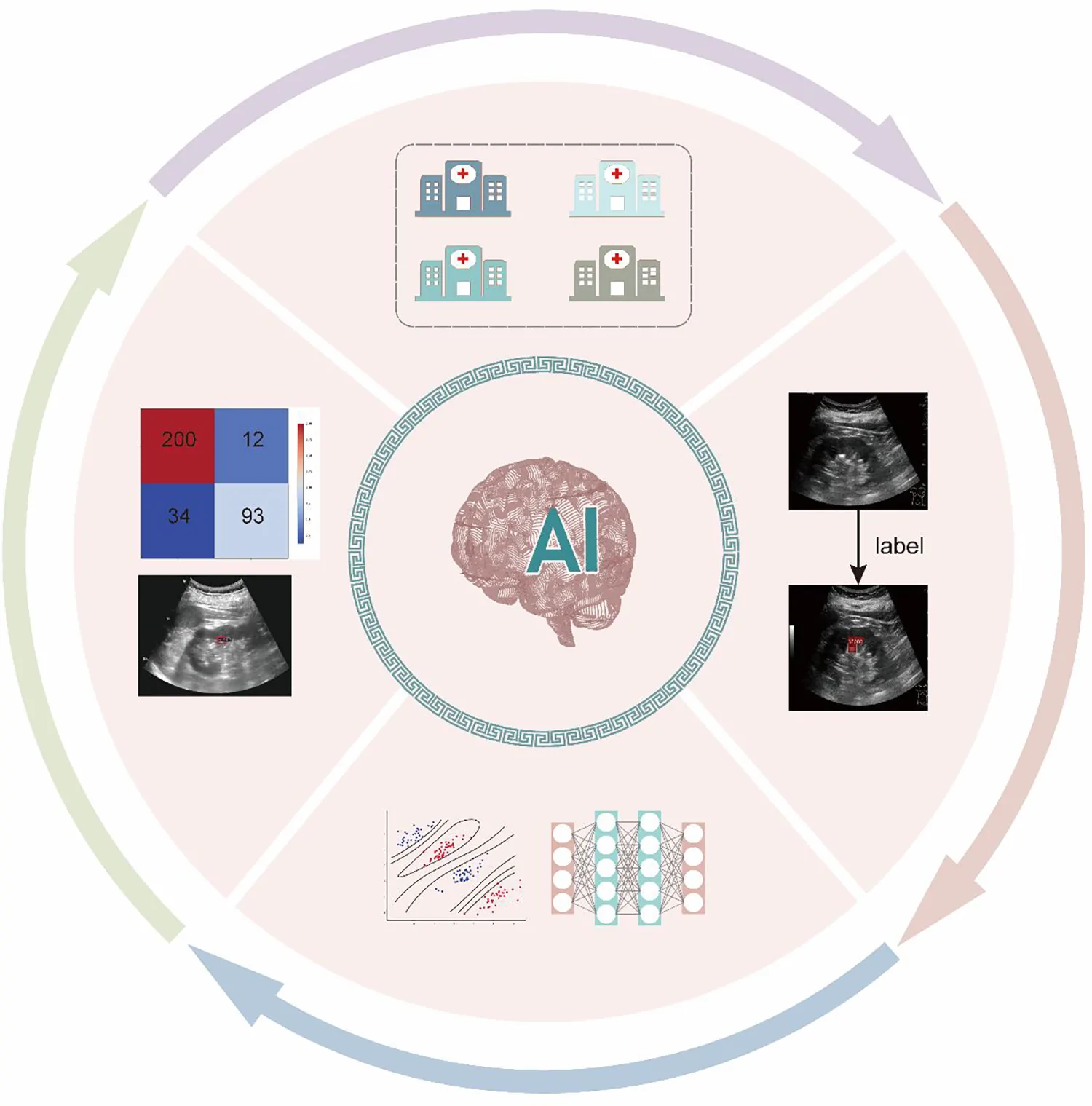
The overall pipeline of the AI model. The diagram demonstrates the process of the AI model from data collection to model establishment. The data is converted into the COCO datasets, and then the HRNET module is applied for training to build the AI model. This model is then used to make judgments with data from additional datasets.

### Data collection

Ultrasound image data were meticulously collected from patients with confirmed renal stones and non-stone participants. The data collection process involved capturing high-quality ultrasound images using standardized protocols to ensure consistency and accuracy. The images were securely stored in a digital repository for subsequent processing and sharing. Advanced data processing techniques were employed to ensure the accuracy and efficiency of medical information, including data cleaning and normalization to reduce noise and enhance image quality.

### Ultrasound image acquisition

All ultrasound examinations were performed using by certified sonographers following a standardized scanning protocol. Images were acquired in both longitudinal and transverse planes with the patient in supine and/or flank positions. Image acquisition parameters (including gain, depth, and frequency settings) were recorded but not standardized across all examinations to reflect real-world clinical variability. A total of 1,130 ultrasound images were included in the final dataset.

### Inclusion and exclusion criteria

Inclusion criteria for stone patients: (1) age ≥18 years; (2) ultrasound-confirmed diagnosis of at least one renal stone; (3) availability of complete ultrasound image data. Exclusion criteria: (1) poor image quality (defined as inability to clearly visualize renal structures due to technical factors such as excessive noise, motion artifact, or inadequate penetration); (2) prior history of renal surgery that could alter renal anatomy; (3) concurrent renal malignancy or other significant renal pathology that could confound stone detection.

### Dataset composition and partitioning

The initial dataset comprised 1,180 participants. After excluding 50 samples with poor ultrasound quality, 1,130 images remained. Following a data audit to resolve inconsistencies in the original records, the final dataset was determined to contain 918 stone images and 212 non-stone images. Images from the same patient were not split across different sets to prevent data leakage. Patient-level identifiers were used to ensure that all images from a given patient were assigned exclusively to one of the sets.

The training/validation set contained 791 stone images. The independent test cohort contained 127 stone images and 212 non-stone images, totaling 339 images. The test cohort corresponds exactly to the reported confusion matrix (TN = 200, FP = 12, FN = 34, TP = 93), which yields 212 actual negatives and 127 actual positives, as shown in the confusion matrix in Figure 4.

### Data labeling

To enhance the readability and practicality of the data, the professional image annotation tool named labelme, was utilized to conduct detailed annotations on the critical regions of the images. This process involved identifying and marking the location and size of renal stones within the images. Annotations were performed by experienced medical professionals to ensure accuracy. This step not only provided intuitive visual references for researchers but also supplied essential data support for subsequent analyses and the training of AI models.

### Data exploration and pre-processing

The annotated data were transformed into the COCO (Common Objects in Context) format to align with the specifications of diverse systems and algorithms. COCO is a widely-adopted format for object detection and image segmentation, known for its standardized data structure. This format facilitated seamless integration of the data into various analysis and processing systems, enhancing the data's versatility and unlocking new avenues for research on the diagnosis and treatment of kidney stones. Data exploration involved analyzing the distribution of stone sizes, locations, and image quality to identify any potential biases or limitations in the datasets.

### Feature selection

Feature selection was a critical step in preparing the data for AI model training. Relevant features that could aid in the detection of renal stones were extracted. These features included stone size, shape, echogenicity, and surrounding tissue characteristics. The selection process involved both manual feature extraction and automated feature selection techniques to identify the most informative features for model training.

### Machine learning models

The High-Resolution Network (HRNet) was selected as the primary machine learning model for this study (22). HRNet is designed to maintain high-resolution representations throughout the entire process, making it particularly suitable for tasks requiring detailed image analysis (23). HRNet achieves cross-resolution information interaction by parallelly connecting multi-resolution subnetworks and repeatedly exchanging information among them (24). This architecture enables HRNet to maintain high-resolution feature representations and perform multi-scale feature fusion, significantly enhancing the efficiency and accuracy of the detection process (25, 26).

Stage 1 begins with a single high-resolution branch, processing the input image through convolutional layers to generate a detailed feature map. Stage 2 introduces a second branch with lower resolution, capturing coarser features. These two branches exchange information, allowing the high-resolution branch to benefit from coarser details and vice versa. Stage 3 adds a third branch with even lower resolution, continuing the information exchange among all three branches to ensure a comprehensive multi-scale representation. Stage 4 may include additional lower-resolution branches, maintaining the principle of cross-resolution interaction. This architecture ensures that HRNet captures both fine-grained and coarser features, enhancing its ability to perform detailed image analysis. This design is particularly effective for tasks requiring high accuracy and robustness, such as medical imaging, where maintaining detailed representations is crucial for tasks like detecting renal stones in ultrasound images.

A high-resolution feature pyramid network (HRFPN) was used as the neck. The input channels of the HRFPN were 18, 36, 72, and 144, and the output channel was 256. The detector contained a region proposal network (RPN) and a standard RoI head. The RPN used anchor ratios of 0.5, 1.0, and 2.0, an anchor scale of 8, and feature strides of 4, 8, 16, 32, and 64. The RPN classification loss was sigmoid cross-entropy, and the bounding-box regression loss was L1 loss. The RPN training assigner used positive IoU 0.7, negative IoU 0.3, minimum positive IoU 0.3, and match_low_quality = True. The RPN sampler used 256 samples with a positive fraction of 0.5. During testing, the RPN used non-maximum suppression (NMS) with an IoU threshold of 0.7, nms_pre=1000, max_per_img=1000, and min_bbox_size=0.

The ROI head was a Standard ROI Head with a Single Extractor. Align was used with an output size of 7 × 7, sampling_ratio=0, feature strides of 4, 8, 16, and 32, and 256 output channels. The bounding-box head was a Shared2FCBBoxHead with 256 input channels, 1024 fully connected output channels, roi_feat_size=7, num_classes=1, and reg_class_agnostic = False. The RoI classification loss was cross-entropy without sigmoid, and the bounding-box regression loss was L1 loss. The RoI training assigner used positive IoU 0.5, negative IoU 0.5, minimum positive IoU 0.5, and match_low_quality = False. The RoI sampler used 512 samples with a positive fraction of 0.25 and add_gt_as_proposals = True. During testing, the ROI head used a classification score threshold of 0.05, NMS with an IoU threshold of 0.5, and max_per_img=100.

The model was trained on the training subset and validated on the validation subset. The final independent evaluation was performed on the test cohort. The detector produced bounding boxes and confidence scores for a single object class, namely renal stone.

The detector was implemented in PyTorch2.0.0/MMDetection 3.3.0 and trained on an NVIDIA RTX 3080 Ti GPU. The backbone was initialized with ImageNet-pretrained HRNetV. Input images were resized with keep-aspect-ratio to 1,333 × 800. The only training augmentation was random horizontal flip with probability 0.5; no vertical flip, rotation, scaling, brightness/contrast jitter, Gaussian blur, or Gaussian noise was used.

The optimizer was SGD with momentum 0.9, weight decay 1e-4, initial learning rate 0.02, and batch size 2. A linear warmup was applied for the first 500 iterations with start_facto*r* = 0.001, followed by MultiStepLR with milestones at epochs 8 and 11 and gamma 0.1. Training ran for 12 epochs, with validation every epoch. No early stopping was used.

During inference, RPN NMS IoU was 0.7, nms_pre=1000, max_per_img=1000, and min_bbox_size=0. The RoI head used NMS IoU 0.5, max_per_img=100, and score_th*r* = 0.05.

At the image level, an image was classified as stone-positive if at least one ROI had a classification score ≥ 0.05. This threshold was inherited from the detector configuration and was not separately optimized on the validation set; this is a limitation.

### Probability estimate stratification

In multiple stages of HRNet, multiple lower-resolution image features were gradually combined to improve the proportion and thickness of high-resolution feature maps. This process enhanced the model's ability to capture fine-grained details and improve detection accuracy. The optimal combination of image features was determined through iterative training and validation. Finally, all feature maps were combined proportionally to generate the final high-resolution image, which was used to estimate the probability of the presence of renal stones. The flowchart is shown in Figure 2.

### Statistical methods

For binary classification tasks, accuracy was calculated as the fraction of correct predictions in all predictions:

$$\mathrm{Accuracy}=\frac{TP+TN}{TP+FP+FN+TN}$$

Where TP is true positive, TN is true negative, FP is false positive, and FN is false negative. The confusion matrix, which has four cells (TP, FN, FP, TN), was also used to evaluate the performance of the model. Sensitivity and specificity were calculated as follows:

$$\mathrm{Sensitivity}=\frac{TP}{FN+TP}$$

$$\mathrm{Specifity}=\frac{TN}{FP+TN}$$

Positive predictive value (PPV) and negative predictive value (NPV) were calculated as:

$$\mathrm{PPV}=\frac{TP}{TP+FP}$$

$$\mathrm{NPV}=\frac{TN}{TN+FP}$$

The model showed high specificity but moderate sensitivity.

## Results

The AI model for renal stone detection was evaluated and achieved an accuracy of 86.4% (95% CI 82.4–89.7%) in the independent test cohort, indicating effective overall diagnostic performance. The confusion matrix analysis revealed a sensitivity of 73.2% (95% CI 64.9–82.2%), specificity of 94.3% (95% CI 90.4–96.7%), positive predictive value (PPV) of 88.6% (95% CI 81.1–93.3%), and negative predictive value (NPV) of 85.5% (95% CI 80.4–89.4%). The model therefore showed high specificity but moderate sensitivity.

In the reported confusion matrix, 34 stones were missed and 12 non-stone cases were falsely classified as positive. The false-negative proportion was 26.8% among actual stone cases in the test cohort. These results indicate that the model is better at confirming renal stones when it returns a positive result than at excluding stones when it returns a negative result.

The visualization of renal stones was presented in Figure 3. An ultrasound image of the kidney was displayed in Figure 3A, while the application of AI modules in detecting renal stones was illustrated in Figure 3B. The model detected 93 of 127 stones (73.2%).

**Figure 3:**
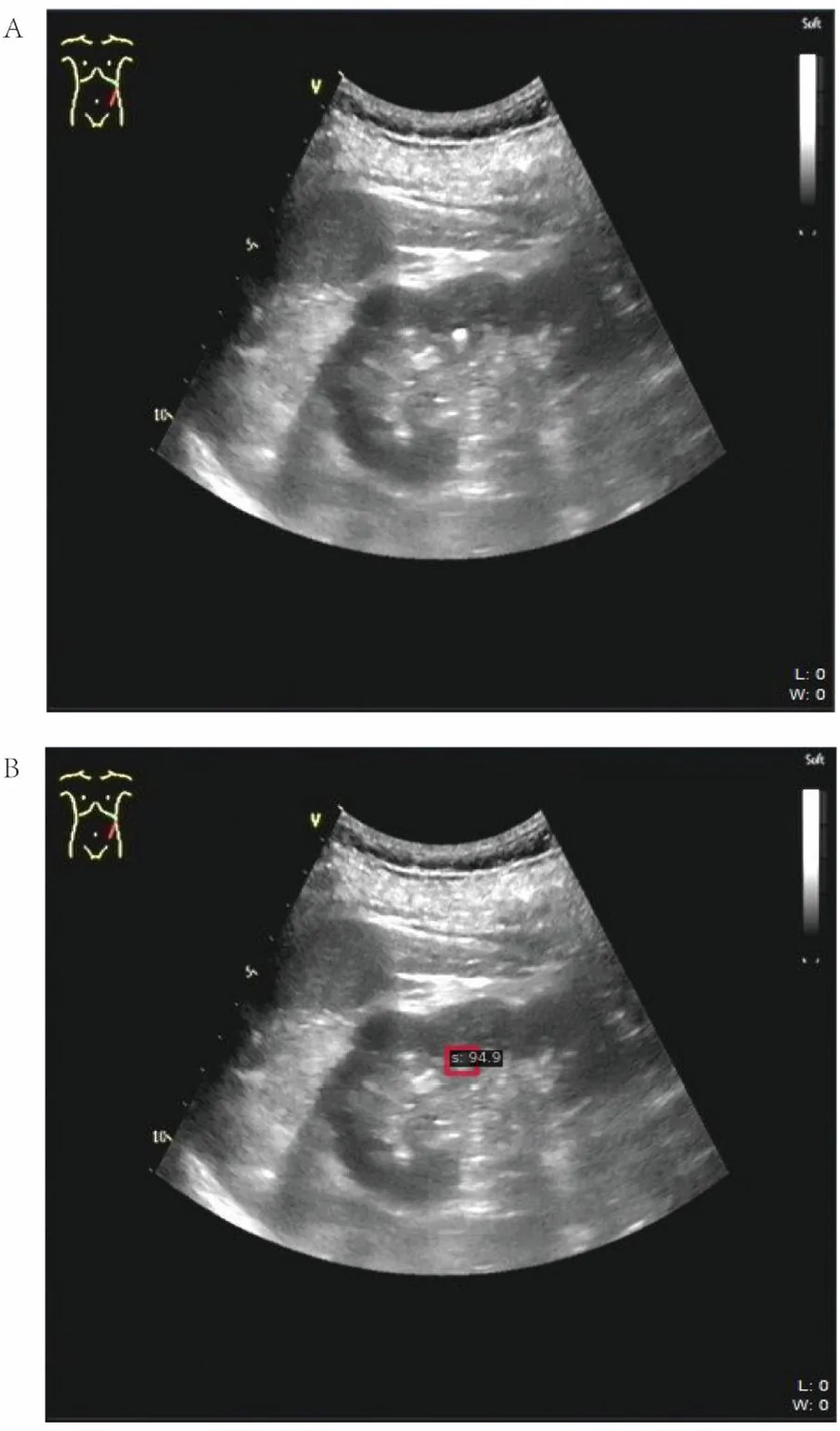
Visualization of renal stone. **(A)** is an ultrasound image, and **(B)** is a visualization of the application of AI modules. The image shows the percentage of stones that can be diagnosed.

The performance of the model in identifying renal stones and non-stone cases was evaluated using the confusion matrix of the test cohort, as shown in Figure 4.

**Figure 4:**
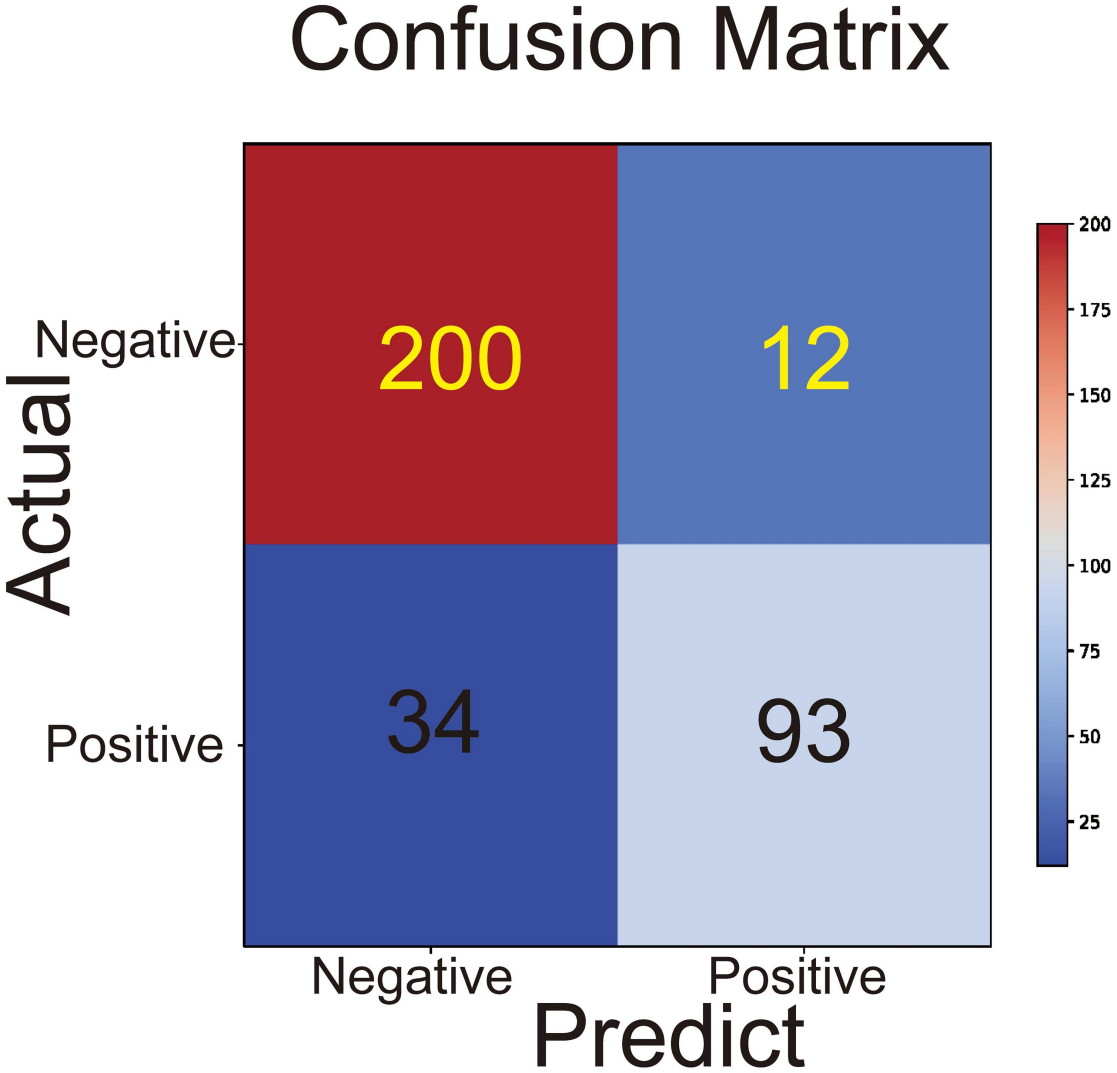
The confusion matrix of the test cohort. The model showed high specificity but moderate sensitivity, missing 34 of 127 stones (26.8%). It cannot currently be used as a stand-alone rule-out or screening tool.

## Discussion

### Significance of AI in renal stone detection

The incorporation of artificial intelligence (AI) into medical diagnostics has shown remarkable potential for enhancing the accuracy and efficiency of renal stone detection (16, 17, 27, 28). Traditional ultrasonic methods, despite their widespread use, are subject to variability based on the experience and skill of the operating physician (6). This variability can lead to inconsistent diagnostic outcomes, especially when influenced by factors such as stone size, location, and surrounding tissues. The application of AI, particularly the High-Resolution Network (HRNet) in this study, addresses these limitations by automating the analysis of ultrasound images and providing consistent image-level detection of renal stones (12, 13, 23, 29–33).

AI's ability to process large volumes of complex data and identify subtle patterns that may escape human interpretation is crucial for renal stone detection (32, 33). The variability in stone characteristics and surrounding tissues can obscure findings, making it challenging to identify renal stones accurately (13, 18).

AI may provide consistent image-level predictions, but this study did not compare AI with human readers (16–18, 33, 34). This consistency is essential in clinical settings, where accurate and timely detection of renal stones is crucial for effective treatment and management (18).

These potential workflow benefits were not evaluated in this retrospective image-level study.providing consistent, potential to improve specificity/consistency detection of renal stones (15, 16, 20, 25, 35). By automating the analysis of ultrasound images, AI models can quickly identify potential renal stones, reducing the time required for diagnosis and allowing clinicians to focus on patient care (13, 19, 27, 31, 32, 36).

Although AI has potential in renal stone detection, the present study only evaluated image-level diagnostic performance. It did not measure clinical workload, diagnostic time, treatment decisions, or patient outcomes. Therefore, claims that this approach reduces diagnostic burden or improves patient outcomes are not supported by the present data.

### Model performance and clinical implications

In the independent test cohort (*n* = 339), the HRNet model achieved an accuracy of 86.4%, sensitivity of 73.2%, specificity of 94.3%, PPV of 88.6%, and NPV of 85.5%. The high specificity indicates that positive results were relatively reliable and that the model may help reduce false-positive findings. However, the moderate sensitivity means that 34 of 127 stones (26.8%) were missed. The NPV of 85.5% also indicates that a negative result still carried a 14.5% risk of missed stone. Therefore, the model is better at rule-in than rule-out and cannot currently be used as a stand-alone diagnostic or screening tool. Further optimization, including training with non-stone images, threshold selection on validation data, and multi-center validation, is required before clinical adoption.

The use of HRNet in this study demonstrates the potential of AI to significantly enhance the diagnostic process. By maintaining high-resolution representations throughout the network, HRNet is able to capture fine-grained details in images, making it particularly well-suited for tasks requiring detailed image analysis. This capability is essential for the accurate detection of renal stones, which can vary in size and shape and may be obscured by surrounding tissues.

### Challenges and future directions

Despite the promising results, several challenges remain in the application of AI for renal stone detection (13, 31). One of the primary issues is the quality and quantity of data required for training and refining AI models (37). Large-scale, annotated datasets are essential for effective learning and validation of AI algorithms (10, 38, 39). The development of such datasets requires significant resources and collaboration between medical professionals and data scientists. Ensuring that AI models can consistently and accurately detect renal stones across a wide range of patient demographics and clinical scenarios is crucial for their widespread adoption.

Another challenge is the need for rigorous testing and validation of AI models to ensure their reliability and stability in real-world applications. The model's performance must be validated on larger and more diverse datasets to ensure its generalizability and clinical applicability. Additionally, the integration of AI into clinical workflows requires careful consideration of ethical and practical implications, including patient privacy and data security.

The dataset was relatively small and derived from a single institution, limiting generalizability. The training/validation set contained no non-stone images, which limited threshold selection and calibration. Future work should include non-stone images during training and validation, use larger multi-center datasets, perform external validation, and report both image-level and patient-level metrics. The decision threshold should be prespecified and selected on validation data according to the intended clinical use. Prospective studies are needed to evaluate workflow integration, diagnostic time, reader performance, patient outcomes, and cost-effectiveness.

### Limitations of the study

This study has several important limitations. First, it was a single-center, retrospective, image-level study. The findings may not generalize to other institutions, ultrasound machines, or patient populations. Second, the independent test cohort was relatively small, especially for stone-positive cases (*n* = 127), which limits the precision of sensitivity estimates. Third, the training/validation set contained no non-stone images, so the image-level threshold could not be optimized on the validation set; the 0.05 threshold was inherited from the detector configuration and is a limitation. Fourth, the model achieved only moderate sensitivity, missing approximately 26.8% of stones in the independent test cohort, which is clinically unacceptable for a stand-alone diagnostic tool. Fifth, no external validation was performed. Sixth, ultrasound image quality depends on the operator, and the study did not compare AI performance with human readers or measure inter-reader variability. Seventh, the study did not evaluate patient outcomes, diagnostic time, clinical workload, treatment decisions, or cost-effectiveness. Finally, the 0.05 threshold was prespecified but was not clinically justified or optimized on validation data.

## Conclusions

HRNet-based AI achieved high specificity but only moderate sensitivity for ultrasound-based renal stone detection, missing approximately one in four stones in the independent test cohort. The model may have potential as an adjunctive rule-in tool after further optimization, but it cannot currently replace clinical judgment or function as a stand-alone screening or diagnostic test. Prospective, multi-center, patient-level studies with prespecified thresholds and clinically meaningful outcomes are required before clinical adoption. Claims about improved patient outcomes or reduced physician workload are not supported by this retrospective image-level study.

## Data Availability

The original contributions presented in the study are included in the article/Supplementary Material, further inquiries can be directed to the corresponding author.

## References

[1] Yao L Yang P. Relationship between remnant cholesterol and risk of kidney stones in U.S. Adults: a 2007-2016 NHANES analysis. Ann Med. (2024) 56(1):2319749. 10.1080/07853890.2024.2319749 38733306 PMC11089921

[2] Ferraro PM Taylor EN Curhan GC. 24-Hour Urinary chemistries and kidney stone risk. Am J Kidney Dis. (2024) 84(2):164–9. 10.1053/j.ajkd.2024.02.010 38583757 PMC13170619

[3] Tamborino F Cicchetti R Mascitti M Litterio G Orsini A Ferretti S. Pathophysiology and main molecular mechanisms of urinary stone formation and recurrence. Int J Mol Sci. (2024) 25(5):3075. 10.3390/ijms25053075 38474319 PMC10931946

[4] Xu Z Li Z Guo M Bian H Niu T Wang J. Application of three-dimensional visualization fused with ultrasound for percutaneous renal puncture. Sci Rep. (2021) 11(1):8521. 10.1038/s41598-021-87972-8 33875726 PMC8055667

[5] Ciccarese F Brandi N Corcioni B Golfieri R Gaudiano C. Complicated pyelonephritis associated with chronic renal stone disease. Radiol Med. (2021) 126(4):505–16. 10.1007/s11547-020-01315-7 33245481 PMC8007507

[6] Fontenelle LF Sarti TD. Kidney stones: treatment and prevention. Am Fam Physician. (2019) 99(8):490–6. PMID: 30990297 30990297

[7] Armas-Phan M Tzou DT Bayne DB Wiener SV Stoller ML Chi T. Ultrasound guidance can be used safely for renal tract dilatation during percutaneous nephrolithotomy. BJU Int. (2020) 125(2):284–91. 10.1111/bju.14737 30811835

[8] Raffone A Raimondo D Neola D Travaglino A Raspollini A Giorgi M. Diagnostic accuracy of ultrasound in the diagnosis of uterine leiomyomas and sarcomas. J Minim Invasive Gynecol. (2024) 31(1):28–36.e21. 10.1016/j.jmig.2023.09.013 37778636

[9] Raja AS Pourjabbar S Ip IK Baugh CW Sodickson AD O'Leary M. Impact of a health information technology-enabled appropriate use criterion on utilization of emergency department CT for renal colic. AJR Am J Roentgenol. (2019) 212(1):142–5. 10.2214/AJR.18.19966 30403534 PMC12870738

[10] Dai JC Bailey MR Sorensen MD Harper JD. Innovations in ultrasound technology in the management of kidney stones. Urol Clin North Am. (2019) 46(2):273–85. 10.1016/j.ucl.2018.12.009 30961860 PMC6461360

[11] De Perrot T Hofmeister J Burgermeister S Martin SP Feutry G Klein J. Differentiating kidney stones from phleboliths in unenhanced low-dose computed tomography using radiomics and machine learning. Eur Radiol. (2019) 29(9):4776–82. 10.1007/s00330-019-6004-7 30747299

[12] Längkvist M Jendeberg J Thunberg P Loutfi A Lidén M. Computer aided detection of ureteral stones in thin slice computed tomography volumes using convolutional neural networks. Comput Biol Med. (2018) 97:153–60. 10.1016/j.compbiomed.2018.04.021 29730498

[13] Wood BG Urban MW. Detecting kidney stones using twinkling artifacts: survey of kidney stones with varying composition and size. Ultrasound Med Biol. (2020) 46(1):156–66. 10.1016/j.ultrasmedbio.2019.09.008 31635759 PMC6961807

[14] Ahmed F Abbas S Athar A Shahzad T Khan WA Alharbi M. Identification of kidney stones in KUB x-ray images using VGG16 empowered with explainable artificial intelligence. Sci Rep. (2024) 14(1):6173. 10.1038/s41598-024-56478-4 38486010 PMC10940612

[15] Babajide R Lembrikova K Ziemba J Ding J Li Y Fermin AS. Automated machine learning segmentation and measurement of urinary stones on CT scan. Urology. (2022) 169:41–6. 10.1016/j.urology.2022.07.029 35908740 PMC9936246

[16] Checcucci E Autorino R Cacciamani GE Amparore D De Cillis S Piana A. Artificial intelligence and neural networks in urology: current clinical applications. Minerva Urol Nefrol. (2020) 72(1):49–57. 10.23736/S0393-2249.19.03613-0 31833725

[17] Chen AB Haque T Roberts S Rambhatla S Cacciamani G Dasgupta P. Artificial intelligence applications in urology: reporting standards to achieve fluency for urologists. Urol Clin North Am. (2022) 49(1):65–117. 10.1016/j.ucl.2021.07.009 34776055 PMC9147289

[18] Shabaniyan T Parsaei H Aminsharifi A Movahedi MM Jahromi AT Pouyesh S. An artificial intelligence-based clinical decision support system for large kidney stone treatment. Australas Phys Eng Sci Med. (2019) 42(3):771–9. 10.1007/s13246-019-00780-3 31332724

[19] Yildirim K Bozdag PG Talo M Yildirim O Karabatak M Acharya UR. Deep learning model for automated kidney stone detection using coronal CT images. Comput Biol Med. (2021) 135:104569. 10.1016/j.compbiomed.2021.104569 34157470

[20] Cui Y Sun Z Ma S Liu W Wang X Zhang X. Automatic detection and scoring of kidney stones on noncontrast CT images using S.T.O.N.E. Nephrolithometry: combined deep learning and thresholding methods. Mol Imaging Biol. (2021) 23(3):436–45. 10.1007/s11307-020-01554-0 33108801

[21] Ji Y Hwang G Lee SJ Lee K Yoon H. A deep learning model for automated kidney calculi detection on non-contrast computed tomography scans in dogs. Front Vet Sci. (2023) 10:1236579. 10.3389/fvets.2023.1236579 37799401 PMC10548669

[22] Zhao S Bai Z Meng L Han G Duan E. Pose estimation and behavior classification of jinling white duck based on improved HRNet. Animals (Basel). (2023) 13(18):2878. 10.3390/ani13182878 37760278 PMC10525901

[23] Wu Y Chen X Dong F He L Cheng G Zheng Y. Performance evaluation of a deep learning-based cascaded HRNet model for automatic measurement of x-ray imaging parameters of lumbar sagittal curvature. Eur Spine J. (2023) 33:4104–18. 10.1007/s00586-023-07937-5 37787781

[24] Wang G Zhang S Li J Zhao K Ding Q Tian D. CB-HRNet: a class-balanced high-resolution network for the evaluation of endoscopic activity in patients with ulcerative colitis. Clin Transl Sci. (2023) 16(8):1421–30. 10.1111/cts.13542 37154517 PMC10432877

[25] Ahmed S Hossain T Hoque OB Sarker S Rahman S Shah FM. Automated COVID-19 detection from chest x-ray images: a high-resolution network (HRNet) approach. SN Comput Sci. (2021) 2(4):294. 10.1007/s42979-021-00690-w 34056622 PMC8144280

[26] Bao W Niu T Wang N Yang X. Pose estimation and motion analysis of ski jumpers based on ECA-HRNet. Sci Rep. (2023) 13(1):6132. 10.1038/s41598-023-32893-x 37061550 PMC10105691

[27] Caglayan A Horsanali MO Kocadurdu K Ismailoglu E Guneyli S. Deep learning model-assisted detection of kidney stones on computed tomography. Int Braz J Urol. (2022) 48(5):830–9. 10.1590/s1677-5538.ibju.2022.0132 35838509 PMC9388181

[28] Cellina M Cè M Rossini N Cacioppa LM Ascenti V Carrafiello G. Computed tomography urography: state of the art and beyond. Tomography. (2023) 9(3):909–30. 10.3390/tomography9030075 37218935 PMC10204399

[29] Wang J Sun K Cheng T Jiang B Deng C Zhao Y. Deep high-resolution representation learning for visual recognition. IEEE Trans Pattern Anal Mach Intell. (2021) 43(10):3349–64. 10.1109/TPAMI.2020.2983686 32248092

[30] Sabuncu Ö Bilgehan B Kneebone E Mirzaei O. Effective deep learning classification for kidney stone using axial computed tomography (CT) images. Biomed Tech (Berl). (2023) 68(5):481–91. 10.1515/bmt-2022-0142 37129960

[31] Thapaliya S Brady SL Somasundaram E Anton CG Coley BD Towbin AJ. Detection of urinary tract calculi on CT images reconstructed with deep learning algorithms. Abdom Radiol (NY). (2022) 47(1):265–71. 10.1007/s00261-021-03274-7 34605964

[32] Xing Z Zhu Z Jiang Z Zhao J Chen Q Xing W. Automatic urinary stone detection system for abdominal non-enhanced CT images reduces the burden on radiologists. J Imaging Inform Med. (2024) 37(2):444–54. 10.1007/s10278-023-00946-2 38343222 PMC11031534

[33] Yang B Veneziano D Somani BK. Artificial intelligence in the diagnosis, treatment and prevention of urinary stones. Curr Opin Urol. (2020) 30(6):782–7. 10.1097/MOU.0000000000000820 32941256

[34] Dai JC Johnson BA. Artificial intelligence in endourology: emerging technology for individualized care. Curr Opin Urol. (2022) 32(4):379–92. 10.1097/MOU.0000000000000972 35102080

[35] Kianian R Carter M Finkelshtein I Eleswarapu SV Kachroo N. Application of artificial intelligence to patient-targeted health information on kidney stone disease. J Ren Nutr. (2024) 34(2):170–6. 10.1053/j.jrn.2023.10.002 37839591

[36] Islam MN Hasan M Hossain MK Alam MGR Uddin MZ Soylu A. Vision transformer and explainable transfer learning models for auto detection of kidney cyst, stone and tumor from CT-radiography. Sci Rep. (2022) 12(1):11440. 10.1038/s41598-022-15634-4 35794172 PMC9259587

[37] Elton DC Turkbey EB Pickhardt PJ Summers RM. A deep learning system for automated kidney stone detection and volumetric segmentation on noncontrast CT scans. Med Phys. (2022) 49(4):2545–54. 10.1002/mp.15518 35156216 PMC10407943

[38] Li AY Elliot N. Natural language processing to identify ureteric stones in radiology reports. J Med Imaging Radiat Oncol. (2019) 63(3):307–10. 10.1111/1754-9485.12861 30720244

[39] Mukherjee P Lee S Elton DC Nakada SY Pickhardt PJ Summers RM. Fully automated longitudinal assessment of renal stone burden on serial CT imaging using deep learning. J Endourol. (2023) 37(8):948–55. 10.1089/end.2023.0066 37310890 PMC10387157

